# Initiation of therapy for obstructive sleep apnea syndrome: a randomized comparison of outcomes of telemetry-supported home-based vs. sleep lab-based therapy initiation

**DOI:** 10.1007/s11325-021-02371-7

**Published:** 2021-05-15

**Authors:** Ingo Fietze, Sebastian Herberger, Gina Wewer, Holger Woehrle, Katharina Lederer, Aline Lips, Leslee Willes, Thomas Penzel

**Affiliations:** 1 Center for Cardiovascular Medicine, Interdisciplinary Center for Sleep Medicine, Charité—Universitätsmedizin Berlin, Luisenstraße 13, 10117 Berlin, Germany; 2 Sleep and Ventilation Center Blaubeuren, Lung Center Ulm, Ulm, Germany; 3 Advanced Sleep Research GmbH, Berlin, Germany; 4 Willes Consulting Group, San Diego, CA USA; 5 Saratov State University, Saratov, Russian Federation

**Keywords:** Obstructive sleep apnea, Automatic positive airway pressure, Continuous positive airway pressure, Compliance, Adherence

## Abstract

**Purpose:**

Diagnosis and treatment of obstructive sleep apnea are traditionally performed in sleep laboratories with polysomnography (PSG) and are associated with significant waiting times for patients and high cost. We investigated if initiation of auto-titrating CPAP (APAP) treatment at home in patients with obstructive sleep apnea (OSA) and subsequent telemonitoring by a homecare provider would be non-inferior to in-lab management with diagnostic PSG, subsequent in-lab APAP initiation, and standard follow-up regarding compliance and disease-specific quality of life.

**Methods:**

This randomized, open-label, single-center study was conducted in Germany. Screening occurred between December 2013 and November 2015. Eligible patients with moderate-to-severe OSA documented by polygraphy (PG) were randomized to home management or standard care. All patients were managed by certified sleep physicians. The home management group received APAP therapy at home, followed by telemonitoring. The control group received a diagnostic PSG, followed by therapy initiation in the sleep laboratory. The primary endpoint was therapy compliance, measured as average APAP usage after 6 months.

**Results:**

The intention-to-treat population (ITT) included 224 patients (110 home therapy, 114 controls); the per-protocol population (PP) included 182 patients with 6-month device usage data (89 home therapy, 93 controls). In the PP analysis, mean APAP usage at 6 months was not different in the home therapy and control groups (4.38 ± 2.04 vs. 4.32 ± 2.28, *p* = 0.845). The pre-specified non-inferiority margin (NIM) of 0.3 h/day was not achieved (*p* = 0.130); statistical significance was achieved in a post hoc analysis when NIM was set at 0.5 h/day (*p* < 0.05). Time to APAP initiation was significantly shorter in the home therapy group (7.6 ± 7.2 vs. 46.1 ± 23.8 days; *p* < 0.0001).

**Conclusion:**

Use of a home-based telemonitoring strategy for initiation of APAP in selected patients with OSA managed by sleep physicians is feasible, appears to be non-inferior to standard sleep laboratory procedures, and facilitates faster access to therapy.

## Introduction

Obstructive sleep apnea (OSA) is a common disorder, occurring at rate of up to 72% in the general population [1–3]. In addition to excessive daytime sleepiness, the presence of OSA also increases the risk of developing a number of important comorbidities, including hypertension, atrial fibrillation, coronary artery disease, stroke, diabetes and possibly even cancer [4–10].

Continuous positive airway pressure (CPAP) is the gold standard of treatment for OSA. The American Academy of Sleep Medicine (AASM) guidelines recommend CPAP or APAP for all patients with an apnea-hypopnea index (AHI) of ≥15/h, and for symptomatic patients when AHI is ≥5/h [11]. The benefits of CPAP therapy on OSA symptoms, concomitant conditions and quality of life cannot be realized if adherence to therapy is not achieved [12]. Adherence to CPAP therapy has therefore been widely studied, but there are many patient-related and patient-independent factors that influence long-term compliance [13–17].

The current clinical practice for starting CPAP or APAP therapy usually involves in-lab device initiation and titration. Laboratory-based polysomnography (PSG) is labor intensive and comparatively expensive, which could be a barrier for some patients. In addition, the high and increasing prevalence of OSA means that demand often exceeds capacity in sleep laboratories, resulting in long wait times [18]. Furthermore, access to sleep laboratories in some areas may be limited or non-existent.

Home-based respiratory polygraphy (PG) has been shown to be a reliable and cost-effective alternative to PSG for diagnosing sleep apnea [19–23] and is recommended by the AASM in patients without significant comorbidities [24]. Given the high prevalence of sleep apnea in Germany [3] and the long wait times for PSG at sleep laboratories, extending the use of PG and outpatient CPAP titration supported by telemonitoring managed by sleep physicians could provide a faster and easier access to therapy for selected patients and ease the burden of excessive demand at sleep centers.

This study investigated PAP therapy compliance of OSA patients, who had auto-titrating continuous positive airway pressure (APAP) therapy initiated in their home environment and were monitored by a homecare provider via telemonitoring, compared with the current practice of management including therapy initiation in a sleep laboratory and standard follow-up.

## Subjects and methods

### Study design

This randomized, open-label, parallel-group study was conducted at a single center in Germany with patient screening conducted over the period December 2013 to November 2015. The study protocol was approved by the institutional ethics committee. All patients gave written informed consent to participate in the trial, which was conducted in accordance with Good Clinical Practice and the Declaration of Helsinki.

### Patients

Male and female patients aged 18–80 years who had moderate to severe OSA confirmed by ambulatory PG were eligible for the study. PG recordings were scored manually by a board-certified sleep physician. Exclusion criteria were as follows: mask intolerance; disability preventing independent nocturnal device usage; previous treatment for OSA (including uvulopalatopharyngoplasty surgery); modified Mallampati score of T3 or T4; participation in a clinical study within the previous 4 weeks; history of psychiatric, neurological or psychological conditions (excluding stable depression); clinically relevant lung or cardiovascular disease (excluding well-controlled hypertension, and treated cardiac arrhythmias or coronary heart disease); neuromuscular disease; history of cancer in the last 5 years; drug/alcohol abuse; and central sleep-related breathing disorders.

### Intervention groups

Eligible patients who showed moderate to severe OSA (AHI ≥15/h) on 6-channel polygraphy (PG) and met all study inclusion criteria were randomized in a 1:1 ratio to the home management group or the standard of care group. The home therapy group received APAP initiation at home followed by telemetric monitoring; APAP pressure was set to 5–15 mbar for all patients.

The control group had APAP therapy setup and initiation in a sleep laboratory. In this group, patients underwent diagnostic PSG (1 night) and PSG-managed PAP therapy initiation (1–2 nights) in the sleep laboratory; PSG nights (two or three) occurred consecutively. Initial APAP pressure in the sleep laboratory was 5–15 mbar, which could be adjusted individually.

Telemetric monitoring in the home therapy group consisted in a weekly review of the wirelessly transmitted device usage statistics, including usage time, AHI (as measured by the device) and mask leakage by a specialist nurse in the homecare provider’s data center. When usage data indicated problems, patients were contacted by phone by a service team member, who was also accessible to patients via a telephone hotline.

### Outcome measures

The primary endpoint was average APAP usage (hours per night) at 6 months. Usage data for the past 6 months was retrieved from the device memory card. Secondary endpoints were the percentage of days with an APAP usage of ≥4 h and changes from baseline to 6 months in the following: Epworth Sleepiness Scale (ESS) score; total Functional Outcomes of Sleep Questionnaire (FOSQ) score; total Pittsburgh Sleep Quality Index (PSQI) score; and the apnea-hypopnea index (AHI). Additional outcome measures were oxygen desaturation index (ODI); numbers of obstructive, central and mixed events; and mean oxygen saturation (SpO_2_) and leak.

### Assessments

Device usage was assessed and questionnaires completed at baseline, after 2 weeks of APAP therapy, and after 6 months of therapy. Respiratory parameters were determined using PG in both groups before study inclusion and after 6 months of APAP in both groups. In addition, the home management group was evaluated 2 weeks after treatment initiation. Patients in the control group underwent PSG at baseline and during APAP titration.

### Statistical analysis

All analyses were conducted in the intention-to-treat (ITT) population (all patients randomized to treatment) and/or the per-protocol (PP) population (all patients who completed the 6-month follow-up visit and provided APAP usage data) using SAS version 9.4. Descriptive statistics (mean, median, standard deviation [6], standard error, minimum and maximum) were calculated for all continuous variables. Frequencies and percentages were calculated for categorical data. Baseline demographic and clinical characteristics were compared between treatment groups using descriptive statistics and compared using a 2-sided *t* test for continuous parameters and Fisher’s exact test for categorical parameters. Data was controlled for normal distribution prior to further analysis, where applicable.

The primary endpoint (mean nightly APAP usage at 6 months) was assessed with a non-inferiority test by comparing the 2 treatment groups for all patients in the PP population using a non-inferiority 2-sample *t* test at a one-sided significance level of *α* = 0.025. The planned non-inferiority margin (NIM) was −0.3 h/day; a NIM of −0.5 h/day was considered after a publication by the AASM highlighting that 0.5 h would be the margin for a clinically significant difference. [24] In addition, the between-group difference in mean usage was calculated along with the associated 95% confidence interval for the difference. A sensitivity analysis was conducted in a similar fashion on the ITT population using a NIM of −0.5 h/day and assigning missing usage values to zero as a worst-case scenario. A subgroup analysis using ANOVA was conducted in the PP population to assess differences in mean APAP usage between treatment groups at 6 months in the following patient subgroups: by age (≤50 vs. >50 years); male versus female; moderate OSA (baseline AHI 15/h to <30/h) versus severe OSA (baseline AHI ≥30/h); and baseline ESS score ≥13 versus baseline ESS score <13. Each ANOVA model included treatment group, patient subgroup and the interaction term as independent effects. *p* values for the effect of the interaction between treatment group and subgroup are reported for each subgroup analysis.

All secondary analyses were generated as follows: device usage results at 6 months, including the proportion of days used, the proportion of days with usage ≥4 h, mean usage and AHI were compared between groups in the PP population using a 2-sided *t* test. The mean change from baseline to 6 months in ESS score, FOSQ score and PSQI score was compared using a 2-sided *t* test, analyzing patients completing both baseline and 6-month questionnaires. A two-sided significance level of 0.05 was used for all secondary analyses.

## Results

**Fig. 1:**
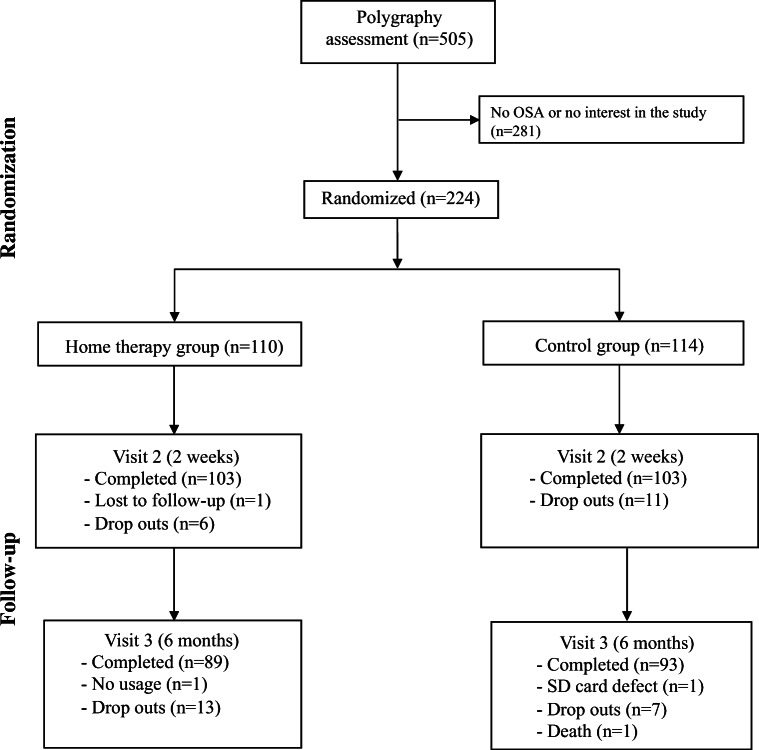
Study flow chart. Completed = having some device usage registered

Of 505 patients initially screened, 224 patients with confirmed OSA were randomized to the home therapy (*n* = 110) or control (*n* = 114) groups and were included in the ITT population (Fig. 1). The PP population included 182 patients who had device usage reported at the 6-month visit (89 in the home therapy group and 93 in the control group) (Fig. 1); completion rates were similar in the two groups (80.9% in the home therapy group and 81.6% in the control group). Four patients in the home therapy group and three in the standard of care group stopped using APAP but completed one or more of the sleepiness and sleep quality questionnaires. Seven patients in the home therapy group and eleven in the standard of care group discontinued the study between randomization and the week 2 visit, and another fourteen in the home therapy group and ten in the standard of care group discontinued between week 2 and 6 months. Two patients required a switch to CPAP or bilevel positive airway pressure. Patient clinical and demographic data at baseline are shown in Table 1. The home therapy and control groups were similar at baseline. Mean follow-up time was 194.6 ± 38.6 days (range 62–336) in the home therapy group and 186.6 ± 36.3 (range 112–384) in the control group.

**Table 1:** Patient demographic and clinical characteristics at baseline (intention-to-treat population)

	Home therapy group (*n* = 110)	Control group (*n* = 114)	*p* value
Age, years	53.6 ± 11.8 (24–80)	53.1 ± 10.6 (28–73)	0.75
Male, *n* (%)	90 (83)	90 (79)	0.50
Caucasian ethnicity, *n* (%)	110 (100)	113 (99)	1.00
BMI, kg/m^2^	32.8 ± 6.4 (21.7–51.7)	31.7 ± 5.6 (21.3–49.5)	0.17
AHI, /h	35.3 ± 17.6 (15.1–110.0)	37.0 ± 20.3 (15.4–108.5)	0.50
ODI, /h	29.9 ± 18.6 (0.3–92.1)	34.5 ± 21.8 (3.8–112.0)	0.09
Mask type, *n* (%)			0.22
Face	19 (17.3)	29 (25.4)	
Nasal	48 (43.6)	39 (34.2)	
Nasal pillows	43 (39.1)	46 (40.4)	
Mean SpO_2_, %	91 ± 3 (72–95)	90 ± 3 (78–95)	0.53
Minimum SpO_2_, %	76 ± 9 (51–91)	74 ± 9 (51–86)	0.08
ESS score	11.2 ± 5.2 (1–24)	10.9 ± 4.9 (2–24)	0.64
PSQI score	9.3 ± 9.8 (0–91)	8.1 ± 3.4 (2–16)	0.22
FOSQ score	87.8 ± 21.9 (11–120)	86.8 ± 20.0 (22–120)	0.74

Values are mean ± standard deviation (range), or number of patients (%)

*AHI* apnea-hypopnea index, *BMI* body mass index, *ESS* Epworth Sleepiness Scale, *FOSQ* Functional Outcomes of Sleep Questionnaire, *PSQI* Pittsburg Sleep Quality Index, *SpO*_*2*_ oxygen saturation

### Device usage

In the PP population, mean APAP device usage at 6 months was similar in the home therapy and control groups at just over 4 h/day (4.38 ± 2.04 vs. 4.32 ± 2.28, respectively, *p* = 0.845). Statistical significance for the initially pre-defined non-inferiority margin of −0.3 h/day was not achieved (*p* = 0.130). However, given the later published clinically important difference of 0.5 h as an important compliance difference by the AASM [25], statistical significance for better compliance in the home management group was achieved for a NIM of −0.5 h/day (*p* = 0.041) (Table 2). In a sensitivity analysis based on the ITT population imputing missing values as the worst case (0 usage) and using a NIM of 0.5 h/day, statistical significance was not achieved (*p* = 0.067) (Table 2). Overall, we did not find any advantage of one treatment approach over the other across any pre-defined patient subgroup analyzed (Table 3).

**Table 2:** Device usage analysis at 6 months

Visit 3 (6 months)	Home therapy group	Control group	Between-group difference (h/day)	*p* value
	Usage (h/day)	Usage (h/day)		
PP population	(*n* = 89)	(*n* = 93)		
Mean ± SD (95% CI)	4.38 ± 2.04 (3.95, 4.81)	4.32 ± 2.28 (3.85, 4.79)	0.06 ± 2.17 (−0.57, 0.70)	
Median (range)	4.40 (0.10–9.58)	4.58 (0.00–8.07)		
NIM −0.3 h/day				0.130
NIM −0.5 h/day				0.041
Sensitivity analysis* ITT population	(*n* = 110)	(*n* = 114)		
Mean ± SD (95% CI)	3.54 ± 2.52 (3.07, 4.02)	3.52 ± 2.66 (3.03, 4.02)	0.02 ± 2.59 (−0.66, 0.70)	
Median (range)	3.76 (0.0–9.6)	3.85 (0.0–8.1)		
NIM −0.5 h/day				0.067

*Missing mean usage imputed as zero

*CI* confidence interval, *h* hours, *ITT* intention-to-treat, *NIM* non-inferiority margin, *PP* per-protocol, *SD* standard deviation

**Table 3:** Device usage at 6 months in patient subgroups (per-protocol population)

Patient subgroup	Home therapy group	Control group	*p* value*
	Usage (h/day)	Usage (h/day)	
Age ≤50 years	(*n* = 31) 4.25 ± 2.11 (3.48, 5.03)	(*n* = 38) 4.08 ± 2.34 (3.32, 4.85)	0.76
Age >50 years	(*n* = 58) 4.45 ± 2.02 (3.92, 4.98)	(*n* = 55) 4.48 ± 2.25 (3.87, 5.09)	
Male	(*n* = 75) 4.47 ± 2.07 (3.99, 4.94)	(*n* = 76) 4.43 ± 2.18 (3.94, 4.93)	0.91
Female	(*n* = 14) 3.93 ± 1.93 (2.81, 5.05)	(*n* = 17) 3.80 ± 2.71 (2.40, 5.19)	
Moderate OSA	(*n* = 44) 3.91 ± 1.97 (3.31, 4.51)	(*n* = 44) 4.26 ± 2.04 (3.64, 4.88)	0.20
Severe OSA	(*n* = 45) 4.84 ± 2.03 (4.23, 5.45)	(*n* = 49) 4.37 ± 2.50 (3.65, 5.09)	
Baseline ESS score <13	(*n* = 56) 4.33 ± 2.12 (3.77, 4.90)	(*n* = 55) 4.40 ± 2.39 (3.76, 5.05)	0.62
Baseline ESS score ≥13	(*n* = 31) 4.46 ± 2.00 (3.72, 5.19)	(*n* = 35) 4.18 ± 2.14 (3.45, 4.92)	

Values are mean ± standard deviation (95% confidence interval)

*ANOVA *p* value for the interaction effect of the subgroup × treatment group

### Secondary endpoints

Device data at 6 months showed similar usage in terms of proportion of days used and proportion of days with usage >4 h in the home therapy and control groups (Table 4). Respiratory values were also similar in both groups and indicated good control of sleep-disordered breathing (Table 3). Sleepiness and sleep quality at 6 months were not significantly different in the two groups (Table 5). Mean time to initiation of APAP therapy was significantly shorter in the home therapy group (7.6 ± 7.2 days, range 0–40 days) compared with the control group (46.1 ± 23.8 days, range 1–119 days), *p* < 0.0001.

**Table 4:** Device usage and respiratory parameters at 6 months (secondary endpoints; per-protocol population)

	Home therapy group	Control group	*p* value
Device data at 6 months	(*n* = 89)	(*n* = 93)	
Total days used, %	79 ± 24 (73.6, 83.7)	74 ± 29 (68, 79)	0.19
Days with usage >4 h, %	61 ± 30 (55, 67)	60 ± 33 (52, 67)	0.75
Respiratory events, /h	1.8 ± 1.9 (1.4, 2.2)	2.0 ± 2.3 (1.5, 2.4)	0.55
95% leakage, L/min	15.6 ± 11.6 (13.1, 18.0)	17.9 ± 35.0 (10.7, 25.1)	0.54
AHI, /h	3.7 ± 4.6 (2.8, 4.7)	5.1 ± 8.6 (3.4, 6.9)	0.17
ODI, /h	3.5 ± 4.7 (2.5, 4.5)	5.7 ± 10.3 (3.6, 7.9)	0.06
OAI, /h	6.0 ± 14.6 (2.9, 9.1)	6.2 ± 17.9 (2.5, 9.9)	0.94
CAI, /h	3.4 ± 5.5 (2.2, 4.5)	4.9 ± 10.2 (2.8, 7.1)	0.20
MAI, /h	0.4 ± 1.3 (0.1, 0.6)	0.8 ± 3.3 (0.2, 1.5)	0.19
HI, /h	20.8 ± 32.2 (13.9, 27.6)	27.0 ± 46.0 (17.5, 36.5)	0.29
Mean SpO_2_, %	93 ± 5 (92, 94)	93 ± 2 (92, 93)	0.73
Minimum SpO_2_, %	84 ± 6 (82, 85)	82 ± 8 (81, 84)	0.21
Mean APAP pressure, cmH_2_O	8.3 ± 2.2 (7.8, 8.7)	8.5 ± 2.0 (8.1, 8.9)	0.57
95% APAP pressure, cmH_2_O	11.2 ± 2.3 (10.7, 11.7)	12.7 ± 10.4 (10.6, 14.9)	0.16

Values are mean ± standard deviation (95% confidence interval)

*AHI* apnea-hypopnea index, *CAI* central apnea index, *HI* hypopnea index, *MAI* mixed apnea index, *OAI* obstructive apnea index, *ODI* oxygen desaturation index

**Table 5:** Change from baseline to 6 months in sleepiness and sleep quality (for all patients who had both baseline and 6-month data available)

	Home therapy group	Control group	Between-group difference in change from baseline	*p* value
ESS score	(n = 90) −2.8 ± 4.4 (−3.7, −1.9)	(n = 92) −3.5 ± 4.7 (−4.5, −2.5)	0.7 ± 4.5 (−0.6, 2.0)	0.28
PSQI score	(n = 90) −2.4 ± 5.6 (−3.6, −1.3)	(n = 93) −2.5 ± 3.4 (−3.2, −1.8)	0.1 ± 4.6 (−1.3, 1.4)	0.93
FOSQ score	(n = 90) 10.7 ± 22.1 (6.1, 15.3)	(n = 89) 6.1 ± 24.5 (0.9, 11.2)	4.7 ± 23.3 (−2.2, 11.5)	0.18

Values are mean ± standard deviation (95% confidence interval)

## Discussion

This study found that device usage, control of sleep-disordered breathing and sleep quality as well as quality of life did not differ when sleep physicians managed APAP therapy at home with ongoing telemonitoring compared with standard in-laboratory, PSG-guided management. Results showed a larger variation in device usage time than expected in the initial sample size calculation. Due to this large variation, the initially defined non-inferiority target of −0.3 h/day could not be proven (Table 2). However, the mean usage in the home therapy was 0.06 h/night higher than in the standard of care group, suggesting that the two approaches were associated with clinically similar levels of APAP device usage after 6 months, despite the fact that statistical significance based on the initially pre-defined NIM of −0.3 h/night was not achieved. A between-group mean difference in a device usage of >0.5 h/night has been defined as clinically significant [25], and non-inferiority was demonstrated in the PP population using −0.5 h/night as the NIM.

In both groups, changes from baseline in the ESS and FOSQ scores also exceeded thresholds defined as being clinically significant [25], highlighting the effectiveness of CPAP therapy in OSA from a patient perspective. It should also be noted that patients in the home therapy group received telemedicine care from the homecare provider throughout the entire study period. This is important because limiting telemedicine-based care (e.g. to 90 days) is associated with a reduction in compliance as has been shown previously [26].

Our study systematically evaluated home therapy with APAP managed by sleep physicians in Germany for the first time. It was also the first time that telemedical care was performed in this setting. Similar outcomes in terms of sleepiness and quality of life with APAP care provided in the home setting versus sleep laboratories have been reported in a meta-analysis [27], consistent with our findings.

One interesting finding of the study was that the time to therapy was significantly shorter using the home-based therapy initiation pathway, which has been reported previously [28]. In our study, patients in the home therapy group received APAP therapy initiation, on average, more than a month sooner than those in the control group, who went to a sleep lab for up to 2 subsequent PSG nights for treatment initiation, in line with the German standards for OSA therapy. The clinical relevance of this remains to be determined. However, earlier diagnosis and therapy of OSA should be beneficial for patients in terms of alleviating OSA symptoms and any potential negative health consequences associated with sleep-disordered breathing. The decrease in waiting times was even greater (up to 6 months) in another recent study that compared a home- versus hospital-based approach to the management of suspected OSA and found the ambulatory approach to be non-inferior to hospital management [28]. The home-based management group also showed a significant improvement in the Sleep Apnoea Quality of Life Index score compared with the standard hospital approach, suggesting that the ambulatory option was better for patients in terms of improved disease-specific quality of life. Another important finding of the study was substantially lower mean costs per patient in the home- versus hospital-based group (HK $8479 vs. HK $22,248) [28]. In addition to the savings achieved by eliminating PSG, both diagnostic and therapeutic, it has also been suggested that nursing time is reduced when a telemonitoring approach is used [29].

There is a growing body of evidence suggesting that PG can be used instead of PSG for the diagnosis and management of OSA. In a study conducted at seven AASM-accredited sleep centers, a home- and PG-based strategy for the diagnosis of OSA and titration/management of APAP therapy was not inferior to in-laboratory PSG with respect to acceptance, adherence, time to treatment and functional improvements at 1 and 3 months [30]. Several other studies have reported similar findings that point towards viability and non-inferiority of the ambulatory treatment initiation [31–36].

The ability of home-based diagnosis and management of OSA holds the potential to improve the efficiency of OSA management, both in terms of healthcare costs and improving access to care [24, 28]. From a US payer perspective, use of PG and APAP has been shown to be significantly less costly than in-lab sleep study and PSG titration [21]. In addition, implementation of a sleep telemedicine protocol improved management of sleep apnea over a period of 5 years, despite an increased demand for services [24]. Data from other studies have also highlighted cost benefits with the use of home respiratory PG management compared with PSG, with similar effectiveness and equivalent or better adherence [20, 36]. It has been suggested that PSG is probably not necessary for the majority of patients with suspected sleep apnea, with a clear economic benefit from the use of home-based management approaches [36]. Nevertheless, it is important to note that the ambulatory approach in our study was managed by sleep physicians and that it is not suitable for all OSA patients. All studies in this field have a variety of exclusion criteria, including unstable comorbidity, other sleep disorders, central sleep apnea, mental illness, drugs, pretreated sleep apnea and others. Therefore, the need for further diagnostic assessment by a certified sleep physician including PSG remains for patients who meet these criteria.

Overall, our findings demonstrate the non-inferiority of the home therapy approach for the diagnosis and management of moderate and severe OSA (AHI >15/h). The results point to a number of potential advantages, both for the healthcare system [21, 28, 36] and for patients (e.g. faster access to care) [28]. In addition to supporting these existing data, our study extends previous work because we used a follow-up period of 6 months, at least twice as long as in earlier studies.

Several limitations of our study can be pointed out:

By applying an AHI of >15/h as inclusion criterion, patients with mild OSA were not included in the investigation. This should be taken into account when applying these results to the general population. The mild OSA group represents a significant part of the symptomatic OSA population and therefore should be addressed in future investigations.

The study design was aimed to demonstrate non-inferiority of a home-based approach in comparison to conventional sleep lab-based APAP treatment initiation with regards to treatment adherence; it did not specifically investigate differences in cost, workload or other measures between the groups. The workload during the 6 months of telemetric follow-up for the home therapy group was not precisely measured but was retrospectively estimated at up to 2 h per patient-month. Meanwhile, the sleep lab group did not receive any structured follow-up after their initial therapy initiation of up to 2 consecutive PSG nights. Future studies are encouraged to investigate details of potential cost reductions and efficacy gains associated with home-based therapy of OSA.

In conclusion, use of a home-based telemonitoring strategy for initiation of APAP therapy by sleep physicians in selected patients with OSA is feasible and comparable to a standard approach with respect to device usage, respiratory parameters, and sleep quality, as well as disease-specific quality of life after 6 months of therapy. In addition, the home-based approach may facilitate faster and easier access to diagnosis and effective therapy in patients with suspected OSA.
